# 2-(4,5-Dihydro-1*H*-imidazol-2-yl)­pyridine

**DOI:** 10.1107/S1600536809009131

**Published:** 2009-03-19

**Authors:** Reza Kia, Hoong-Kun Fun, Hadi Kargar

**Affiliations:** a X-ray Crystallography Unit, School of Physics, Universiti Sains Malaysia, 11800 USM, Penang, Malaysia; b Department of Chemistry, School of Science, Payame Noor University (PNU), Ardakan, Yazd, Iran

## Abstract

In the mol­ecule of the title compound, C_8_H_9_N_3_, a new imidazoline derivative, the six- and five-membered rings are slightly twisted away from each other, forming a dihedral angle of 7.96 (15)°. In the crystal structure, neighbouring mol­ecules are linked together by inter­molecular N—H⋯N hydrogen bonds into extended one-dimensional chains along the *a* axis. The pyridine N atom is in close proximity to a carbon-bound H atom of the imidazoline ring, with an H⋯N distance of 2.70 Å, which is slightly shorter than the sum of the van der Waals radii of these atoms (2.75 Å). The crystal structure is further stabilized by inter­molecular C—H⋯π and π–π inter­actions (centroid-to-centroid distance 3.853 Å).

## Related literature

For related structures and synthesis, see: Stibrany *et al.* (2004 ▶); Kia *et al.* (2008 ▶, 2009*a* ▶,*b* ▶). For biological and pharmaceutical applications, see, for example: Blancafort (1978 ▶); Chan (1993 ▶); Vizi (1986 ▶); Li *et al.* (1996 ▶); Ueno *et al.* (1995 ▶); Corey & Grogan (1999 ▶). For the stability of the temperature controller used for data collection, see: Cosier & Glazer (1986 ▶). For standard bond-length data, see: Allen *et al.* (1987 ▶).
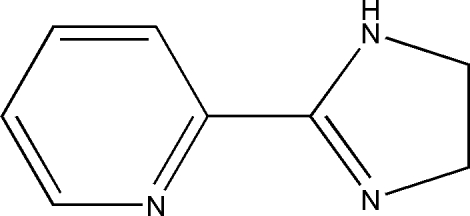


## Experimental

### 

#### Crystal data

C_8_H_9_N_3_
*M* *_r_* = 147.18
Orthorhombic, 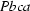

*a* = 10.0057 (8) Å
*b* = 7.9828 (7) Å
*c* = 17.6199 (14) Å
*V* = 1407.4 (2) Å^3^
*Z* = 8
Mo *K*α radiation
μ = 0.09 mm^−1^
*T* = 100 K
0.48 × 0.46 × 0.09 mm

#### Data collection

Bruker SMART APEXII CCD area-detector diffractometer
Absorption correction: multi-scan (*SADABS*; Bruker, 2005 ▶) *T*_min_ = 0.959, *T*_max_ = 0.992
10642 measured reflections
1238 independent reflections
869 reflections with *I* > 2σ(*I*)
*R*_int_ = 0.094

#### Refinement

*R*[*F* ^2^ > 2σ(*F* ^2^)] = 0.051
*wR*(*F* ^2^) = 0.146
*S* = 1.08
1238 reflections
104 parameters
H atoms treated by a mixture of independent and constrained refinement
Δρ_max_ = 0.26 e Å^−3^
Δρ_min_ = −0.31 e Å^−3^

### 

Data collection: *APEX2* (Bruker, 2005 ▶); cell refinement: *SAINT* (Bruker, 2005 ▶); data reduction: *SAINT*; program(s) used to solve structure: *SHELXTL* (Sheldrick, 2008 ▶); program(s) used to refine structure: *SHELXTL*; molecular graphics: *SHELXTL*; software used to prepare material for publication: *SHELXTL* and *PLATON* (Spek, 2009 ▶).

## Supplementary Material

Crystal structure: contains datablocks global, I. DOI: 10.1107/S1600536809009131/wn2314sup1.cif

Structure factors: contains datablocks I. DOI: 10.1107/S1600536809009131/wn2314Isup2.hkl

Additional supplementary materials:  crystallographic information; 3D view; checkCIF report

## Figures and Tables

**Table 1:** Hydrogen-bond geometry (Å, °) *Cg*1 is the centroid of the N3/C4–C8 (pyridine) ring.

*D*—H⋯*A*	*D*—H	H⋯*A*	*D*⋯*A*	*D*—H⋯*A*
N1—H1N1⋯N2^i^	0.85 (3)	2.27 (3)	3.084 (3)	160 (3)
C2—H3⋯*Cg*1^ii^	0.99	2.87	3.611 (3)	133
C6—H6⋯*Cg*1^iii^	0.95	2.84	3.561 (3)	134

Symmetry codes: (i) 
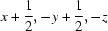
; (ii) 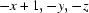; (iii) 
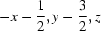
.

**Fig. 1.**
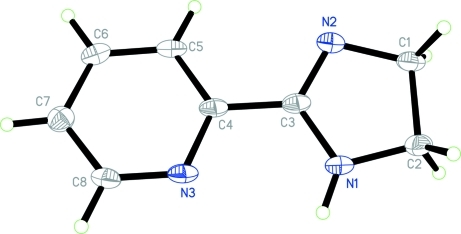
The molecular structure of the title compound, with atom labels. Displacement ellipsoids are drawn at the 50% probability level. H atoms are shown as spheres of arbitrary radius.

**Fig. 2.**
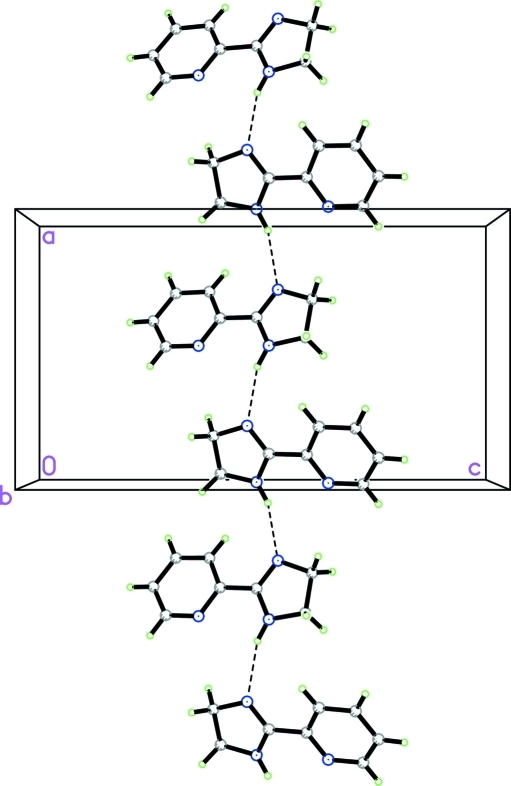
The crystal packing of the title compound, viewed down the b axis, showing a one-dimensional extended chain along the a axis, formed by intermolecular N—H···N interactions. The intermolecular interactions are shown as dashed lines.

## supplementary crystallographic information

### Comment

Imidazoline derivatives are of great importance because they exhibit significant biological and pharmacological activities, such as antihypertensive (Blancafort, 1978), antihyperglycemic (Chan, 1993), antidepressant (Vizi, 1986), antihypercholesterolemic (Li et al., 1996) and anti-inflammatory (Ueno et al., 1995) properties. These compounds are also used as catalysts and synthetic intermediates in some organic reactions (Corey & Grogan, 1999). With regard to these important applications of imidazolines, we report here the crystal structure of the title compound.

In the title compound (Fig. 1), bond lengths (Allen et al., 1987) and angles are within the normal ranges and are comparable with those in related structures (Stibrany et al., 2004; Kia et al., 2008, 2009a,b). The molecule is almost planar, with a maximum deviation from the mean plane of the molecule for atom N1: 0.106 (2) Å. The six- and five-membered rings are twisted from each other, forming a dihedral angle of 7.96 (15)°. Atom H1 of the imidazoline ring is in close proximity to atom N3 of the pyridine ring, with a distance of 2.70 Å [N3···H1], which is shorter than the sum of the van der Waals radii of these atoms (2.75 Å). In the crystal structure, neighbouring molecules are linked together by intermolecular N—H···N hydrogen bonds into one-dimensional extended chains along the a axis (Table 1, Fig. 2). The crystal structure is further stabilized by intermolecular C—H···π [Cg1 is the centroid of the N3/C4–C8 pyridine ring] and π–π interactions [Cg1···Cg2 = 3.853 Å and Cg2 is the centroid of the N1/C1/C2/N2/C3 ring].

### Experimental

The synthetic method was based on previous work (Stibrany *et al.,* 2004), except that 10 mmol of 2-cyanopyridine and 40 mmol of ethylenediamine were used. Single crystals suitable for X-ray diffraction were obtained by evaporation of a methanol solution at room temperature.

### Refinement

The N-bound H atom was located in a Fourier difference map and refined freely (Table 1). The other H atoms were positioned geometrically and refined with a riding approximation model; C—H = 0.95–0.99 Å and *U*_iso_(H) = 1.2*U*_eq_(C).

### Crystal data

**Table:**

C_8_H_9_N_3_	*F*(000) = 624
*M**_r_* = 147.18	*D*_x_ = 1.389 Mg m^−^^3^
Orthorhombic, *P**b**c**a*	Mo *K*α radiation, λ = 0.71073 Å
Hall symbol: -P 2ac 2ab	Cell parameters from 3233 reflections
*a* = 10.0057 (8) Å	θ = 3.1–30.9°
*b* = 7.9828 (7) Å	µ = 0.09 mm^−^^1^
*c* = 17.6199 (14) Å	*T* = 100 K
*V* = 1407.4 (2) Å^3^	Plate, colourless
*Z* = 8	0.48 × 0.46 × 0.09 mm

### Data collection

**Table:**

Bruker SMART APEXII CCD area-detector diffractometer	1238 independent reflections
Radiation source: fine-focus sealed tube	869 reflections with *I* > 2σ(*I*)
graphite	*R*_int_ = 0.094
φ and ω scans	θ_max_ = 25.0°, θ_min_ = 2.3°
Absorption correction: multi-scan (*SADABS*; Bruker, 2005)	*h* = −11→11
*T*_min_ = 0.959, *T*_max_ = 0.992	*k* = −9→9
10642 measured reflections	*l* = −20→20

### Refinement

**Table:**

Refinement on *F*^2^	Primary atom site location: structure-invariant direct methods
Least-squares matrix: full	Secondary atom site location: difference Fourier map
*R*[*F*^2^ > 2σ(*F*^2^)] = 0.051	Hydrogen site location: inferred from neighbouring sites
*wR*(*F*^2^) = 0.146	H atoms treated by a mixture of independent and constrained refinement
*S* = 1.08	*w* = 1/[σ^2^(*F*_o_^2^) + (0.0658*P*)^2^ + 1.1427*P*] where *P* = (*F*_o_^2^ + 2*F*_c_^2^)/3
1238 reflections	(Δ/σ)_max_ < 0.001
104 parameters	Δρ_max_ = 0.26 e Å^−^^3^
0 restraints	Δρ_min_ = −0.31 e Å^−^^3^

### Special details

**Table:**

Experimental. The crystal was placed in the cold stream of an Oxford Cyrosystems Cobra open-flow nitrogen cryostat (Cosier & Glazer, 1986) operating at 100.0 (1) K.
Geometry. All e.s.d.'s (except the e.s.d. in the dihedral angle between two l.s. planes) are estimated using the full covariance matrix. The cell e.s.d.'s are taken into account individually in the estimation of e.s.d.'s in distances, angles and torsion angles; correlations between e.s.d.'s in cell parameters are only used when they are defined by crystal symmetry. An approximate (isotropic) treatment of cell e.s.d.'s is used for estimating e.s.d.'s involving l.s. planes.
Refinement. Refinement of *F*^2^ against ALL reflections. The weighted *R*-factor *wR* and goodness of fit *S* are based on *F*^2^, conventional *R*-factors *R* are based on *F*, with *F* set to zero for negative *F*^2^. The threshold expression of *F*^2^ > σ(*F*^2^) is used only for calculating *R*-factors(gt) *etc*. and is not relevant to the choice of reflections for refinement. *R*-factors based on *F*^2^ are statistically about twice as large as those based on *F*, and *R*- factors based on ALL data will be even larger.

### Fractional atomic coordinates and isotropic or equivalent isotropic displacement parameters (Å^2^)

**Table:**

	*x*	*y*	*z*	*U*_iso_*/*U*_eq_	
N1	0.4819 (2)	0.2650 (3)	−0.01294 (14)	0.0329 (7)	
N2	0.2746 (2)	0.1657 (3)	−0.03211 (12)	0.0232 (6)	
N3	0.4811 (2)	0.1456 (3)	0.13473 (12)	0.0225 (6)	
C1	0.3126 (3)	0.2543 (4)	−0.10232 (14)	0.0251 (7)	
H1	0.2478	0.3446	−0.1137	0.030*	
H2	0.3149	0.1757	−0.1457	0.030*	
C2	0.4531 (3)	0.3285 (4)	−0.08783 (14)	0.0232 (7)	
H3	0.5188	0.2877	−0.1256	0.028*	
H4	0.4516	0.4525	−0.0886	0.028*	
C3	0.3763 (2)	0.1770 (3)	0.01289 (14)	0.0195 (6)	
C4	0.3779 (3)	0.1036 (3)	0.08982 (14)	0.0202 (6)	
C5	0.2758 (2)	−0.0035 (3)	0.11365 (15)	0.0213 (6)	
H5	0.2050	−0.0326	0.0803	0.026*	
C6	0.2797 (3)	−0.0662 (4)	0.18629 (14)	0.0231 (7)	
H6	0.2106	−0.1378	0.2040	0.028*	
C7	0.3852 (3)	−0.0238 (3)	0.23325 (15)	0.0231 (7)	
H7	0.3904	−0.0658	0.2836	0.028*	
C8	0.4829 (3)	0.0814 (4)	0.20475 (15)	0.0229 (7)	
H8	0.5556	0.1097	0.2369	0.027*	
H1N1	0.555 (3)	0.278 (4)	0.0109 (16)	0.036 (9)*	

### Atomic displacement parameters (Å^2^)

**Table:**

	*U*^11^	*U*^22^	*U*^33^	*U*^12^	*U*^13^	*U*^23^
N1	0.0146 (14)	0.0501 (17)	0.0340 (15)	−0.0094 (12)	−0.0053 (12)	0.0105 (12)
N2	0.0147 (13)	0.0261 (13)	0.0289 (12)	0.0008 (10)	−0.0021 (10)	−0.0009 (9)
N3	0.0105 (11)	0.0244 (13)	0.0326 (13)	0.0018 (10)	−0.0013 (10)	−0.0013 (10)
C1	0.0138 (14)	0.0320 (16)	0.0296 (15)	0.0004 (12)	−0.0023 (12)	0.0006 (12)
C2	0.0146 (14)	0.0260 (15)	0.0290 (15)	0.0005 (12)	0.0007 (11)	−0.0008 (11)
C3	0.0118 (14)	0.0173 (14)	0.0292 (15)	0.0027 (11)	0.0018 (11)	−0.0038 (11)
C4	0.0108 (13)	0.0180 (14)	0.0317 (15)	0.0048 (11)	0.0008 (11)	−0.0028 (11)
C5	0.0102 (15)	0.0204 (15)	0.0334 (15)	0.0005 (11)	−0.0010 (11)	−0.0056 (11)
C6	0.0161 (15)	0.0205 (15)	0.0327 (16)	−0.0009 (12)	0.0048 (12)	−0.0017 (11)
C7	0.0204 (15)	0.0203 (14)	0.0287 (15)	0.0029 (12)	0.0023 (12)	0.0001 (11)
C8	0.0148 (15)	0.0259 (15)	0.0278 (15)	0.0007 (12)	−0.0042 (11)	−0.0040 (12)

### Geometric parameters (Å, °)

**Table:**

N1—C3	1.349 (3)	C2—H4	0.9900
N1—C2	1.442 (4)	C3—C4	1.477 (4)
N1—H1N1	0.85 (3)	C4—C5	1.397 (4)
N2—C3	1.293 (3)	C5—C6	1.374 (4)
N2—C1	1.475 (3)	C5—H5	0.9500
N3—C8	1.336 (3)	C6—C7	1.383 (4)
N3—C4	1.343 (3)	C6—H6	0.9500
C1—C2	1.547 (4)	C7—C8	1.383 (4)
C1—H1	0.9900	C7—H7	0.9500
C1—H2	0.9900	C8—H8	0.9500
C2—H3	0.9900		
			
C3—N1—C2	109.6 (2)	N2—C3—C4	122.9 (2)
C3—N1—H1N1	125 (2)	N1—C3—C4	120.5 (2)
C2—N1—H1N1	126 (2)	N3—C4—C5	122.5 (2)
C3—N2—C1	106.1 (2)	N3—C4—C3	116.7 (2)
C8—N3—C4	117.3 (2)	C5—C4—C3	120.7 (2)
N2—C1—C2	106.2 (2)	C6—C5—C4	118.8 (2)
N2—C1—H1	110.5	C6—C5—H5	120.6
C2—C1—H1	110.5	C4—C5—H5	120.6
N2—C1—H2	110.5	C5—C6—C7	119.3 (3)
C2—C1—H2	110.5	C5—C6—H6	120.4
H1—C1—H2	108.7	C7—C6—H6	120.4
N1—C2—C1	101.4 (2)	C8—C7—C6	118.1 (2)
N1—C2—H3	111.5	C8—C7—H7	121.0
C1—C2—H3	111.5	C6—C7—H7	121.0
N1—C2—H4	111.5	N3—C8—C7	124.0 (2)
C1—C2—H4	111.5	N3—C8—H8	118.0
H3—C2—H4	109.3	C7—C8—H8	118.0
N2—C3—N1	116.5 (2)		
			
C3—N2—C1—C2	−3.2 (3)	N1—C3—C4—N3	7.7 (4)
C3—N1—C2—C1	−2.3 (3)	N2—C3—C4—C5	9.5 (4)
N2—C1—C2—N1	3.3 (3)	N1—C3—C4—C5	−172.6 (2)
C1—N2—C3—N1	1.9 (3)	N3—C4—C5—C6	1.2 (4)
C1—N2—C3—C4	179.9 (2)	C3—C4—C5—C6	−178.6 (2)
C2—N1—C3—N2	0.4 (3)	C4—C5—C6—C7	−1.0 (4)
C2—N1—C3—C4	−177.7 (2)	C5—C6—C7—C8	0.3 (4)
C8—N3—C4—C5	−0.5 (4)	C4—N3—C8—C7	−0.4 (4)
C8—N3—C4—C3	179.3 (2)	C6—C7—C8—N3	0.5 (4)
N2—C3—C4—N3	−170.2 (2)		

### Hydrogen-bond geometry (Å, °)

**Table:**

*D*—H···*A*	*D*—H	H···*A*	*D*···*A*	*D*—H···*A*
N1—H1N1···N2^i^	0.85 (3)	2.27 (3)	3.084 (3)	160 (3)
C2—H3···Cg1^ii^	0.99	2.87	3.611 (3)	133
C6—H6···Cg1^iii^	0.95	2.84	3.561 (3)	134

Symmetry codes: (i) *x*+1/2, −*y*+1/2, −*z*; (ii) −*x*+1, −*y*, −*z*; (iii) −*x*−1/2, *y*−3/2, *z*.
